# Reprogramming Immunosuppressive Niches and the Cancer Immunity Cycle in Pancreatic Cancer with Neoantigen mRNA Plus Immune Adjuvant Nanocarrier Strategies

**DOI:** 10.1021/acsnano.5c14753

**Published:** 2025-11-22

**Authors:** André E. Nel, Lijia Luo, Yu-Pei Liao, Xiang Wang

**Affiliations:** † Division of NanoMedicine, Department of Medicine, David Geffen School of Medicine, University of California, Los Angeles, California 90095, United States; ‡ California NanoSystems Institute, University of California, Los Angeles, California 90095, United States; § Division of NanoMedicine, California Nano Systems Institute, University of California, Los Angeles, California 90095, United States

**Keywords:** immune tolerance, reprogramming, pancreatic ductal adenocarcinoma (PDAC), mRNA, lipid nanoparticle, immunotherapy, tumor microenvironment, immune adjuvant nanocarrier

## Abstract

Pancreatic ductal adenocarcinoma (PDAC) remains among the most lethal malignancies, driven by an immune-excluded, desmoplastic tumor microenvironment (TME), a low neoantigen burden, and resistance to immune checkpoint blockade. Recent progress in immunotherapy has emerged from two complementary fronts—personalized mRNA vaccination and nanomedicine-based immune reprogramming. Clinically, the autogene cevumeran (BNT122) mRNA–lipoplex vaccine demonstrated that individualized neoantigen delivery can elicit durable, tissue-resident CD8^+^ T cells and prolong recurrence-free survival in resected PDAC, marking a breakthrough in restoring adaptive immunity to an otherwise “cold” tumor. Preclinically, multifunctional nanocarriers have expanded this potential to advanced disease: lipid nanoparticles (LNPs) codelivering mutant KRAS G12D mRNA and the STING agonist, cGAMP, reprogram tolerogenic hepatic antigen-presenting cells into immune activators, inducing type I interferon signaling and the ability to eradicate liver metastases in murine PDAC models. This approach overcomes the immune protective niche that promotes metastatic cancer growth in the liver. Complementary silicasome platforms (lipid bilayer coated mesoporous silica nanoparticles) encapsulating irinotecan to induce immunogenic cell death (ICD) synergize with spleen-targeting LNPs carrying KRAS mRNA and TLR7/8 agonists, thereby bridging local antigen release with systemic T-cell priming. Together, these studies establish a translational framework wherein nanoparticle-based immunotherapy can both enhance and extend the benefits of mRNA vaccines from localized to metastatic PDAC. By integrating ICD induction, neoantigen targeting, and immune niche reprogramming, these modular nanomedicine platforms offer a realistic path toward scalable, durable, and systemically effective PDAC immunotherapy.

## Graphical abstract

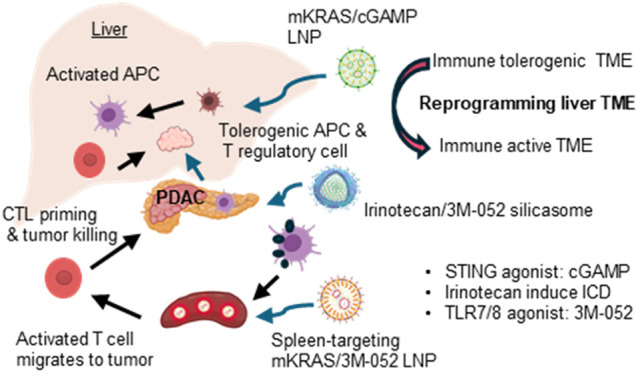

## 1 Introduction

Pancreatic ductal adenocarcinoma (PDAC) remains one of the most lethal malignancies, with a projected rise to the second leading cause of cancer-related death by 2040.^1–3^ Despite major strides in immunotherapy across several cancer types, PDAC has proven stubbornly resistant, with less than 1% of patients responding to immune checkpoint inhibitors.^4,5^ This immune evasion is largely due to PDAC’s profoundly immunosuppressive tumor microenvironment (TME), which includes a fibrotic stroma that restricts immune cell infiltration, a low neoantigen burden, dysfunctional antigen-presenting cells (APCs), and the dominance of regulatory immune cell populations.^6,7^ These features jointly disrupt the cancer immunity cycle, which constitutes a series of steps by which the immune system recognizes and eliminates cancer cells.^8,9^

This focused review seeks to highlight a novel nanomedicine-based strategy to restore the disrupted cancer immunity cycle in PDAC, an undertaking that is urgently required due to the persistent failure of conventional immunotherapy approaches and the aggressive progression of metastatic disease in PDAC.^7,10^ Rather than offering a generalized overview of nanotherapeutics, we concentrate on three recent studies that collectively chart a path forward for effective immunotherapy by combining immunogenic cell death, neoantigen presentation, and immune niche reprogramming through engineered nanoparticles.^11–13^

The first study, from our own laboratory, conducted in a mouse model, demonstrates that lipid nanoparticles (LNPs) delivering both mutant KRAS mRNA and a STING agonist (cGAMP) can reprogram the tolerogenic immune landscape in the liver, a key site for occurrence of PDAC metastasis (Figure 1).^11^ This dual-delivery approach converts liver APCs from immunosuppressive to an immunostimulatory phenotype, inducing Type I interferon responses and generating CD8^+^ cytotoxic T cells capable of tumor eradication. Strikingly, the induced immunity in the animal model is durable and transferable, offering both prophylactic and therapeutic potential.^11^

**Figure 1:**
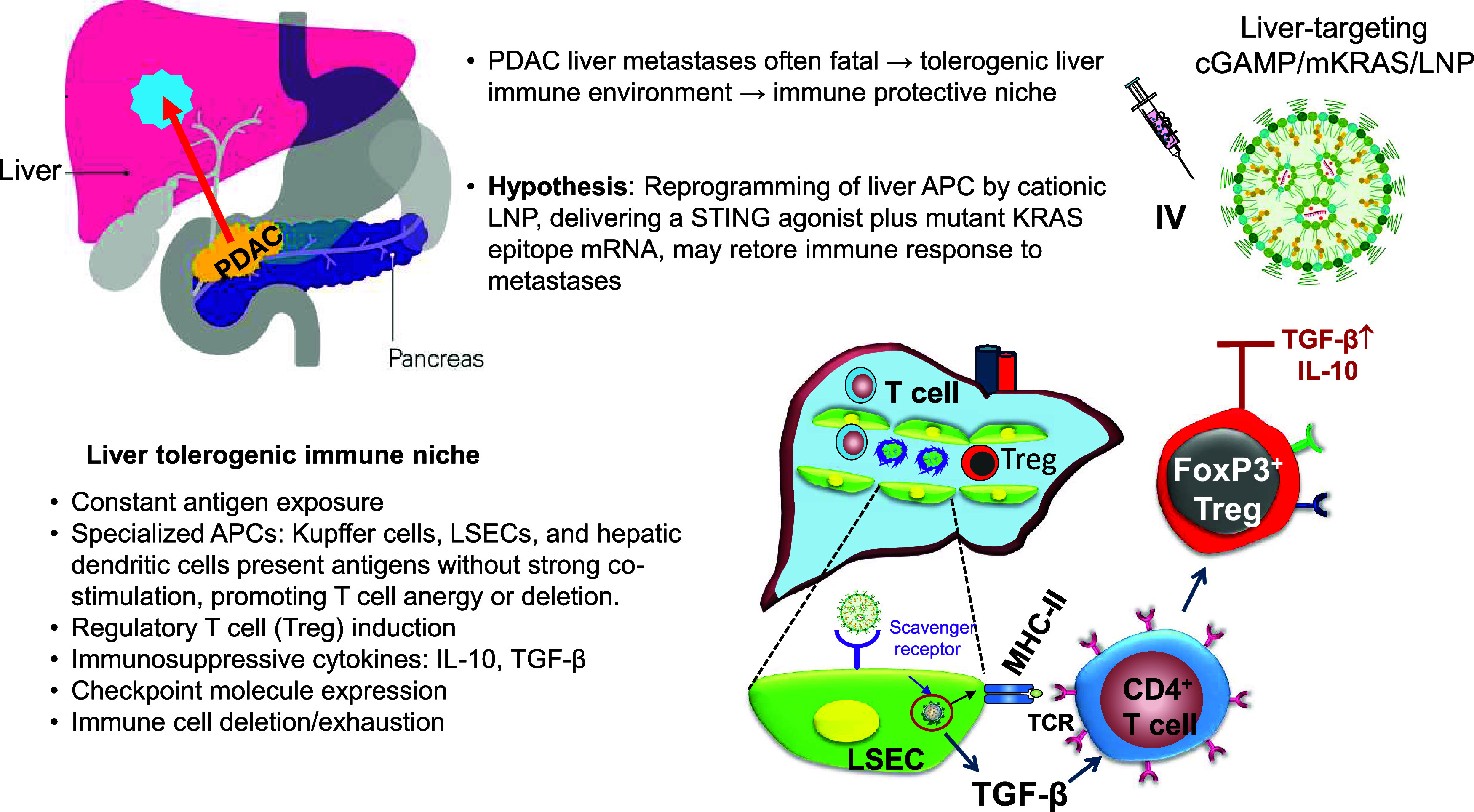
Schematic overview of the rationale for our preclinical study, “Reprogramming the Tolerogenic Immune Response Against Pancreatic Cancer Metastases by Lipid Nanoparticles Delivering a STING Agonist Plus Mutant KRAS mRNA”.^11^ The liver is a frequent site of PDAC metastasis, and presents a major barrier to immunotherapy due to its tolerogenic APCs, including Kupffer cells, liver sinusoidal endothelial cells (LSECs), and dendritic cells. These cells promote regulatory T-cell induction (bottom right diagram), release immunosuppressive cytokines such as IL-10 and TGF-β, and express checkpoint receptors, collectively fostering an immune-privileged niche for metastatic growth. This often leads to failed attempts at PDAC immunotherapy and a high mortality rate. Building on prior findings that hepatic APCs (e.g., LSECs) can uptake and express exogenous mRNA,^74^ we hypothesized that codelivery of a STING agonist (cGAMP) and a mutant KRAS neoantigen epitope via lipid nanoparticles would reprogram the liver APCs from an immunosuppressive to an immunostimulatory state. This strategy aims to trigger a type I interferon–driven immune response, activate CD8^+^ T cells, and ultimately suppress PDAC liver metastases and prolong survival *in vivo* (Figure 4).

The second preclinical study, also authored by us, built on this by combining two distinct nanoparticle systems: silicasomes loaded with irinotecan to induce immunogenic cell death (ICD) at the tumor site, and spleen-targeting LNPs delivering KRASG12D mRNA and the TLR7/8 agonist 3M-052 (Figure 2).^12^ This strategy promotes systemic immune activation by linking tumor antigen release to T-cell priming in the spleen, thus reinforcing the cancer immunity cycle. Silicasome nanocarriers—lipid-bilayer–coated mesoporous silica nanoparticles—have been extensively characterized for their stability, drug-loading versatility, and tumor-targeting efficiency across several gastrointestinal and genitourinary cancers, including pancreatic and bladder malignancies.^14–16^ The dual-platform significantly reduced tumor burden, extended survival, and showed molecular synergy during study of immune gene activation.

**Figure 2:**
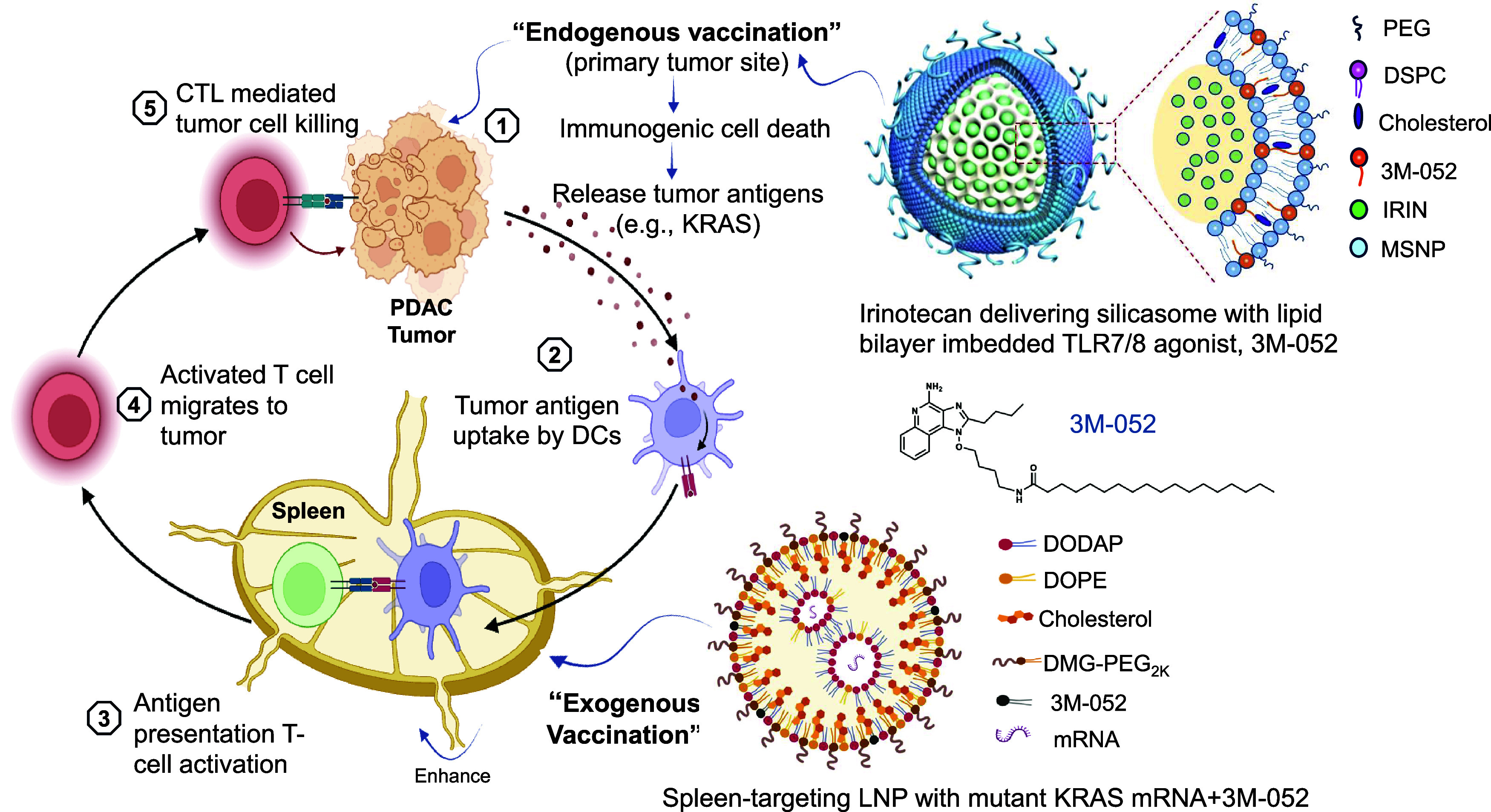
Schematic illustration of our nanocarrier immunotherapy strategy, which hypothesizes that integration of both endogenous and exogenous vaccination responses could be used to enhance the PDAC cancer immunity cycle at the level of the spleen and the primary tumor site.^12^ The cancer immunity cycle proceeds through release of tumor antigens, dendritic cell (DC) presentation, T-cell priming in the spleen, and subsequent cytotoxic T lymphocyte (CTL)–mediated tumor killing. Our strategy employs two distinct nanocarrier platforms to synergistically reinforce CTL activation against PDAC. Endogenous vaccination: A mesoporous silica nanoparticle coated with a lipid bilayer (“silicasome”)^36^ delivers irinotecan together with the TLR7/8 agonist 3M-052. Irinotecan induces immunogenic cell death (ICD), releasing tumor antigens and danger-associated molecular patterns (DAMPs) that activate DCs and prime CTLs in the spleen, while 3M-052 augments innate immunity and APC activation.^40^ Exogenous vaccination: Spleen-targeting LNPs codeliver mutant KRAS mRNA and a TLR7/8 agonist, driving robust generation of tumor-specific CTLs. As shown in Figures 5 and 6, the combined approach synergistically amplifies CTL priming, activation, and trafficking to the tumor site, resulting in enhanced and durable antitumor immunity. Figure adapted with permission from ref 12 under a Creative Commons CC BY 4.0 license. Copyright 2025 The Authors.

The third study, advancing to clinical trial status, comes from Memorial Sloan Kettering Cancer Center, where an individualized mRNA–lipoplex vaccine (autogene cevumeran) targeting patient-specific neoantigens was used in conjunction with surgery, chemotherapy, and checkpoint blockade (Figure 3A).^13,17^ This trial achieved *de novo* generation of mRNA neoantigen-induced CD8^+^ T cells with remarkable longevity and effector function in approximately half of treated patients, with a clear correlation between immune response and recurrence-free survival (Figure 3B).^13^ Yet, this success currently is limited to patients without advanced disease, underscoring the need for strategies that can impact more advanced disease, including reprogramming of immune-resistant metastatic niches. Accordingly, the two preclinical studies referred to above, provide mechanistic insights and translational guidance that could enhance the effectiveness of such vaccines in the metastatic setting, particularly through liver and spleen immune microenvironment reprogramming. This advance reflects the use of nanocarriers, that apart from mRNA delivery, also codeliver immune adjuvant stimuli that reprograms the tolerogenic immune environment as well as engaging the spleen through the cancer immunity cycle.^11,12^

**Figure 3:**
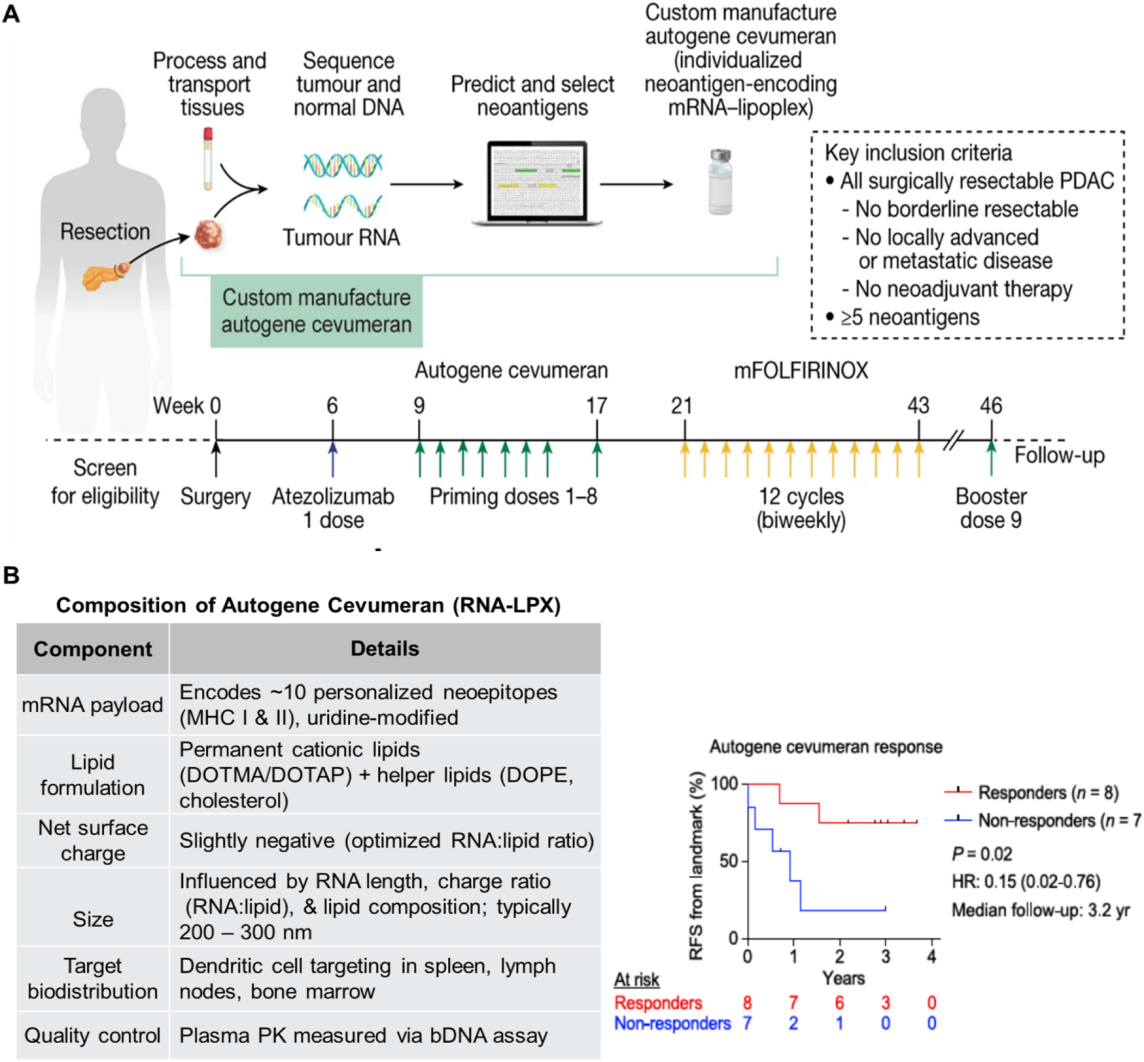
Design and clinical outcome of a personalized mRNA–lipoplex (RNA-LPX) vaccine trial in PDAC, conducted by Sethna et al.,^13^ in follow-up to the previous study of Rojas et al.^17^ (A) Schematic of the phase I trial evaluating autogene cevumeran, an individualized mRNA-based neoantigen vaccine, administered in combination with surgery, FOLFIRINOX chemotherapy, and anti–PD-L1 therapy (atezolizumab) in patients with resected PDAC. Figure reprinted with permission from ref 17 under a Creative Commons CC BY license. Copyright 2023 The Authors. (B) (Left) Composition of the autogene cevumeran RNA-LPX formulation.^75^ (Right) Recurrence-free survival (RFS) following the final vaccine priming dose: patients mounting vaccine-induced T cell responses (“responders”) demonstrated significantly prolonged RFS compared with nonresponders, underscoring the potential of mRNA vaccines to enhance antitumor immunity in PDAC. Figure reprinted with permission from ref 13. Copyright 2025 Springer Nature.

Together, these three studies support a unifying hypothesis that multifunctional nanoparticles can overcome PDAC’s immunologic barriers by simultaneously enhancing antigen availability, reprogramming immune environments in the liver and spleen, and inducing durable, neoantigen-specific T cell responses. Throughout this review, we explore how the integration of these principles could lead to rationally designed, multimodal immunotherapies capable of transforming outcomes for patients with PDAC.

## 2 Neoantigen mRNA Vaccination in PDAC: Breakthroughs and Barriers

Neoantigen mRNA vaccines represent a promising frontier in PDAC immunotherapy, as they harness patient-specific tumor antigens to elicit robust and durable immune responses.^13,17^ A landmark first-in-human phase I trial tested autogene cevumeran—an individualized mRNA-based vaccine also known as BNT122 or RO7198457 in patients with resected PDAC, in combination with surgery, FOLFIRINOX chemotherapy, and anti–PD-L1 therapy (Figure 3A).^13,17^ Autogene cevumeran is an RNA–lipoplex (RNA-LPX) formulation in which encapsulated mRNA encodes up to ten patient-specific neoepitopes (Figure 3B).^13,17,18^ Neoantigen selection involved identifying expressed nonsynonymous mutations and patient HLA alleles through whole-exome sequencing of tumor–normal pairs and RNA sequencing of tumor tissue (Figure 3A). Candidate epitopes were ranked bioinformatically for predicted HLA binding affinity and immunogenicity, ensuring inclusion of the most promising PDAC-specific T-cell stimulatory epitopes.^13,17^ Importantly, the vaccine encoded short peptide epitopes rather than full-length antigens, thereby focusing immune recognition on mutated regions most capable of eliciting strong CD8^+^ T-cell responses.

Among the most frequently selected neoantigens were KRAS codon 12 variants, TP53, and SMAD4, reflecting their high prevalence in PDAC and their suitability as immunogenic targets.^13,17^ Notably, vaccine-induced tissue-resident memory CD8^+^ T-cell responses were detected in 8 of 16 patients, and these “immune responders” exhibited significantly prolonged recurrence-free survival compared to nonresponders (Figure 3B).^13^ Transcriptomic and TCR sequencing revealed that the vaccine primed de novo T-cell responses against previously untargeted passenger mutations rather than simply expanding pre-existing clones.

Interestingly, responders and nonresponders did not differ in the overall number of mutations or candidate neoantigens, but responders’ tumors displayed neoantigens of greater computational quality and higher clonality—features more likely to drive robust T-cell recognition.^13^ Other baseline factors, including tumor burden, immune cell frequencies, or responses to unrelated vaccines, showed no significant differences between the two groups.^13^ Collectively, this study demonstrates that personalized mRNA vaccines can generate long-lived, functionally competent T cells in PDAC, directly correlating with clinical benefit. These findings reinforce the principle that neoantigen quality, rather than quantity, is a key determinant of immunogenic success in low–mutational-burden tumors like PDAC.^5^

While personalized mRNA LNP vaccines offer highly tailored therapeutic potential, their translation into widespread clinical use is limited by long production timelines and high costs.^13,18^ In the autogene cevumeran trial, vaccine manufacturing required approximately 9 weeks from surgery to infusion—a major drawback in PDAC, where disease progression is often rapid. However, recent advances in sequencing technology and computational neoantigen prediction are beginning to mitigate these limitations. Ultrarapid whole-exome and RNA sequencing workflows have now reduced turnaround times to less than 3 weeks, while artificial intelligence–based algorithms such as DeepNeo and pVACtools have improved the precision of epitope prediction, minimizing the need for extensive experimental validation.^19,20^ In parallel, modular mRNA-LNP manufacturing platforms, such as BioNTech’s GMP-automated production systems, enable standardized, scalable synthesis of individualized vaccine batches using shared lipid and RNA components. These innovations collectively promise to shorten vaccine preparation time, lower costs, and increase accessibility. Moreover, combining such technological improvements with semipersonalized vaccine approaches that include shared, recurrent mutations (e.g., KRAS codon 12 variants) could further streamline manufacturing and broaden clinical applicability, as discussed below.

KRAS mutations are found in more than 90% of PDAC cases, with the G12D variant being the most common, occurring in 35–45% of patients (Figure 4).^21–23^ Targeting such prevalent driver mutations offers a practical solution for developing “off-the-shelf” mRNA vaccines, eliminating the need for individualized neoantigen profiling while covering the majority of PDAC patients. Importantly, as will be described in the next subsection, an mRNA vaccine encoding KRAS^G12D^ and delivered via LNPs has been shown to elicit robust immune responses against PDAC.^11^ By prioritizing common KRAS variants, mRNA-LNP vaccines could become more scalable, cost-effective, and broadly applicable, accelerating their integration into clinical practice. These advances also highlight the need for innovative nanomedicine platforms capable of delivering mRNA vaccines and adjuvants directly to metastatic sites, re-establishing the cancer immunity cycle and improving outcomes in advanced PDAC. Building on this rationale, it is important to explore how liver and spleen–targeted nanoparticle systems can reprogram immune-resistant niches and extend vaccine efficacy to metastatic disease.

**Figure 4:**
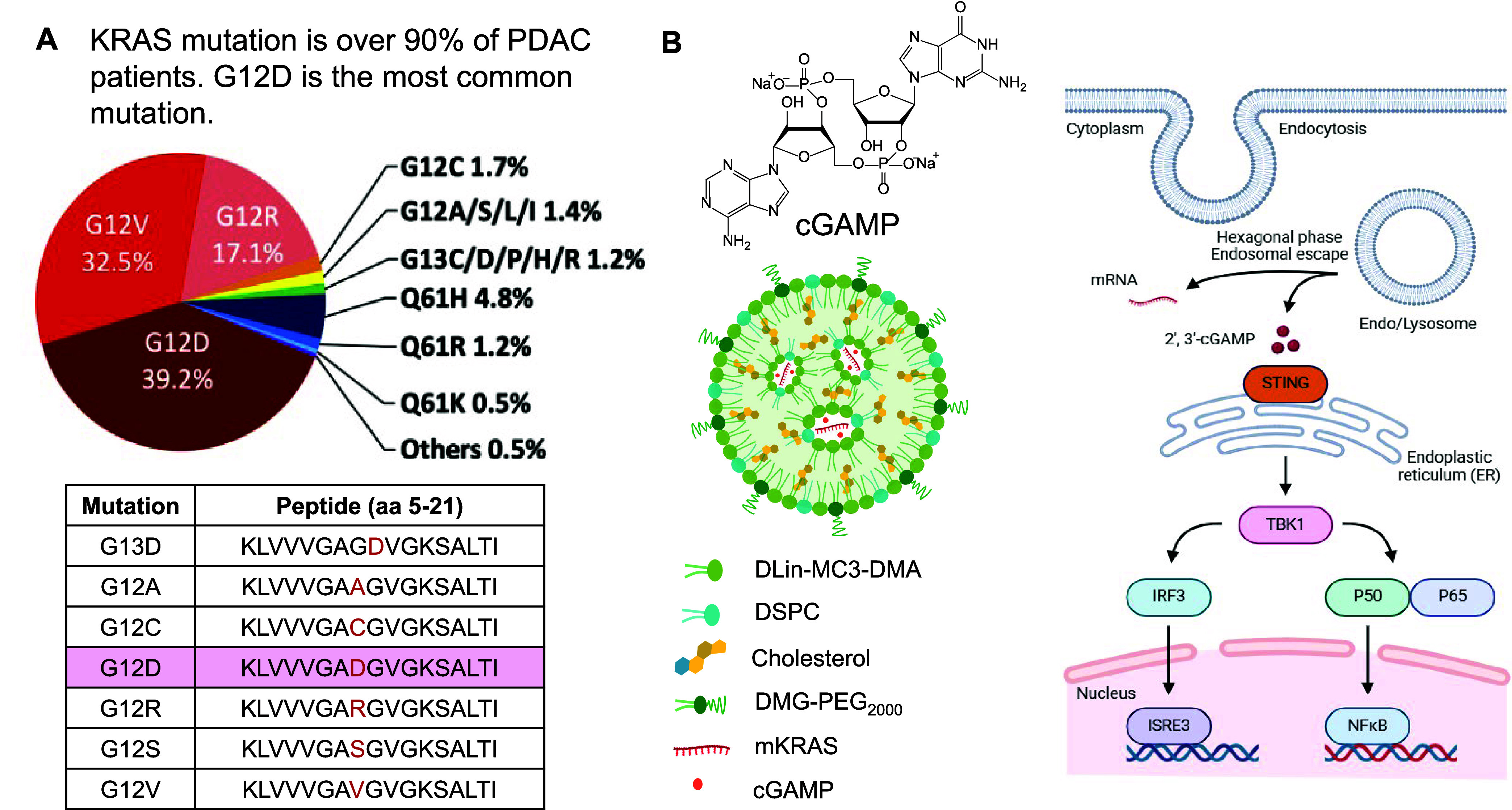
Schematic illustrating the use of a specific KRAS mutation as an immunotherapy target in a murine PDAC liver metastasis model, employing a cationic lipid nanoparticle (LNP) to codeliver KRAS^G12D^ mRNA and the STING agonist cGAMP.^11^ (A) Frequency distribution of KRAS mutations in human PDAC,^42^ with corresponding neoantigen peptide sequences listed in the accompanying table (mutated residues highlighted in red).^11^ KRAS mutations occur in >90% of PDAC cases, with G12D being the single most prevalent variant (35–45% of all cases). Figure reprinted with permission from ref 42. Copyright 2021 Elsevier. (B) Schematic of the KRAS^G12D^ mRNA/cGAMP cationic LNP, which demonstrated predominant liver uptake and generates a vaccine-like immune response by reversing hepatic immune tolerance. cGAMP activates the STING pathway, driving type I interferon production and proinflammatory cytokine release, thereby enhancing antigen presentation and cytotoxic T-cell priming. Figure adapted with permission from ref 11. (https://pubs.acs.org/doi/10.1021/acsnano.4c14102). Copyright 2025 American Chemical Society.

## 3 Reprogramming the Liver Metastatic Niche by a Combination of a STING Agonist Plus mKRAS (KRAS mRNA)

We have already outlined that the liver is a frequent site of PDAC metastases, in part due to the presence of tolerogenic hepatic APCs, including Kupffer cells, liver sinusoidal endothelial cells (LSECs), and dendritic cells (DCs) (Figure 1).^24–30^ A pivotal mouse model for studying PDAC metastasis demonstrated that intravenously administered LNPs, codelivering mRNA encoding the KRAS^G12D^ neoantigen and the STING agonist cGAMP, is capable of reprogramming the tolerogenic liver microenvironment to enhance anti-PDAC immunity (Figure 4).^11^ The rationale for selecting KRAS^G12D^ is that over 90% of oncogenic KRAS mutations in PDAC occur at G12, with KRAS^G12D^ being the most prevalent variant - present in approximately 35–45% of PDAC cases.^31,32^ A potent STING pathway activator, cGAMP, was included to overcome hepatic immune tolerance and augment antitumor immunity (Figure 4).^11,33^ The KRAS/cGAMP nanoparticle was formulated with the ionizable cationic lipid DLin-MC3-DMA [(6*Z*,9*Z*,28*Z*,31*Z*)-heptatriaconta-6,9,28,31-tetraen-19-yl-4-(dimethylamino)­butanoate], which enables efficient endosomal escape and mRNA delivery. Upon systemic administration, the particle acquires a liver-targeting protein corona that drives preferential uptake by hepatic APC, thereby localizing immunostimulatory activity to the tolerogenic liver niche.^11^ This dual payload induced upregulation of costimulatory molecules (CD80/CD86) on liver APCs, triggered Type I interferon signaling, mobilized CD8^+^ cytotoxic T cells (against KRAS), and significantly reduced metastatic burden while extending survival in a KRAS-driven PDAC mouse model.^11^ This approach leverages the prevalence of KRAS mutations and the immunostimulatory potential of STING activation to counteract the liver’s tolerogenic environment, a critical barrier in PDAC metastasis.

Adoptive transfer experiments in the PDAC metastatic liver model further revealed the formation of immunological memory and immune durability.^11^ Mice treated with KRAS^G12D^ mRNA and cGAMP LNPs developed long-lived CD8^+^ T cells with memory phenotypes, protecting against tumor rechallenge. This aligns with observations from neoantigen vaccine trials, where tissue-resident memory T cells correlated with prolonged recurrence-free survival.^13^ Recent studies on PDAC liver metastases have similarly emphasized the importance of durable T-cell responses, noting that combinatorial immunotherapies, such as PD-1 inhibitors with anti-CD137 agonist antibody and GVAX, a whole-cell granulocyte-macrophage colony-stimulating factor (GM-CSF)-secreting allogenic PDAC vaccine, can enhance memory T-cell formation in KRAS-mutated PDAC, though with limited efficacy in advanced metastatic settings.^34^ Our approach of directly targeting liver APC, offers a more tailored solution for establishing immune memory in metastatic PDAC.^11^

Emerging preclinical and clinical studies of PDAC liver metastases underscore the necessity of sustained T-cell immunity. While immune checkpoint blockade (e.g., PD-1 inhibitors) has shown limited efficacy alone in metastatic PDAC due to the immunosuppressive hepatic microenvironment, combination regimens with chemotherapy (such as gemcitabine/nab-paclitaxel plus nivolumab) have modestly improved 1-year survival rates (57.7% vs 35% with chemo alone).^35^ However, objective responses remain sparse in metastatic disease. Importantly, in addition to reprogramming of the liver metastatic niche, there is increasing recognition of the value of chemotherapy in priming antitumor immune responses, including through the ability to induce ICD (Figure 5).^36^ In this regard, we have previously demonstrated that irinotecan delivery by mesoporous silica nanoparticles, decorated with a lipid bilayer, induce a robust ICD response in a KRAS PDAC model.^36^ Irinotecan is a component of the FOLFIRINOX chemotherapy regimen used in the autogene cevumeran study.^17^ This finding sets the stage for the additional preclinical approach discussed in this review, which combines irinotecan-loaded silicasomes with spleen-targeting KRAS mRNA LNPs to integrate local ICD with systemic immune reprogramming.

**Figure 5:**
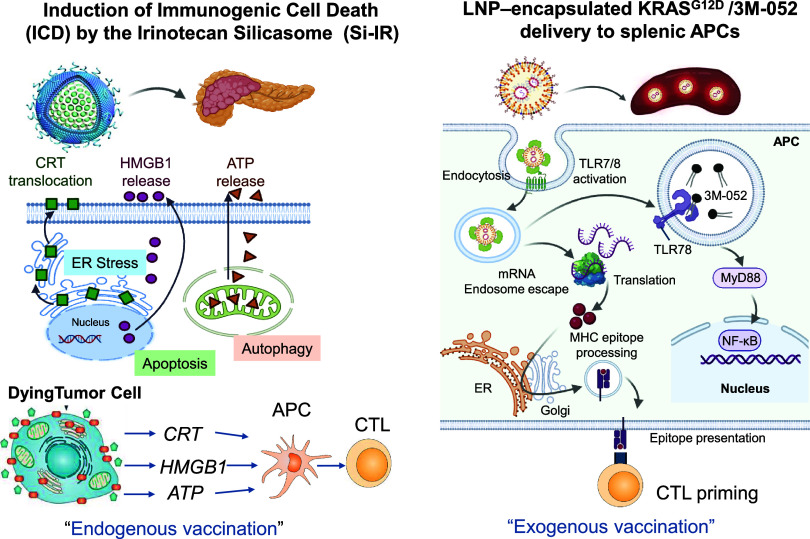
Schematic illustrating the concepts of immunogenic cell death (ICD) and spleen-mediated reinforcement of the cancer immunity cycle through a combination nanocarrier strategy, as outlined in Figure 2.^12^ (Left) Endogenous vaccination is achieved with the irinotecan-loaded silicasome platform, which induces endoplasmic reticulum stress and ICD at the primary tumor site. This programmed death pathway promotes the exposure and release of immunogenic signals, including calreticulin (CRT) and damage-associated molecular patterns (DAMPs) such as HMGB1 and ATP. These mediators facilitate tumor antigen uptake, activate antigen-presenting cells (APCs), and prime cytotoxic T lymphocytes (CTLs). (Right) Exogenous vaccination employs a spleen-targeting cationic LNP codelivering KRAS^G12D^ mRNA and the TLR7/8 agonist, 3M-052. Spleen selectivity is conferred by inclusion of the pH-sensitive cationic lipid DODAP in combination with the helper lipid DOPE. Upon systemic administration, these particles acquire an organ-selective protein corona, undergo endocytosis by splenic APCs, and activate TLR7/8 signaling. Subsequent endosomal escape allows translation of KRAS^G12D^ mRNA into antigenic peptides, which are processed via the MHC I pathway and presented to CTLs, thereby reinforcing tumor-specific adaptive immunity. Figures adapted with permission from ref 12 under a Creative Commons CC BY 4.0 license. Copyright 2025 The Authors.

## 4 Enhancing the Cancer Immunity Cycle: Integrating ICD with Immunotherapy

The cancer immunity cycle, a multistep process critical for effective antitumor immunity, is disrupted in PDAC due to poor tumor immunogenicity and a highly immunosuppressive TME, particularly during antigen release, presentation, and immune cell priming (Figure 5).^5–9^ Integrating ICD inducers with spleen-targeting LNPs, including a KRAS neoantigen epitope, offers a synergistic strategy to reinvigorate the cancer immunity cycle in PDAC.^12,13,37^ In a pivotal study, Luo et al. demonstrated that combining irinotecan-loaded silicasomes with spleen-targeting LNPs encapsulating KRAS^G12D^ mRNA and the toll-like receptor 7/8 (TLR7/8) agonist, 3M-052, can markedly enhance the cancer immunity cycle in an orthotopic PDAC mouse model.^12^ This resulted in significant tumor regression and prolonged survival. The spleen-targeting nanoparticles were engineered using the pH-sensitive cationic lipid DODAP (1,2-dioleoyl-3-dimethylammonium-propane) together with the helper lipid DOPE (1,2-dioleoyl-*sn*-glycero-3-phosphoethanolamine) to form a formulation optimized for endosomal escape and mRNA release. This lipid composition shapes the adsorbed protein corona in the circulation, favoring splenic dendritic cell uptake and thereby enhancing antigen presentation and T-cell priming in the spleen. Autogene cevumeran is also a spleen-targeting mRNA–lipoplex nanocarrier, formulated with ionizable/cationic lipids, helper phospholipids, cholesterol and PEG-lipids.^13,18^

Spleen-targeting exploit this lymphoid organ’s unique microarchitecture for delivering KRAS^G12D^ mRNA and the TLR7/8 agonist 3M-052 directly to splenic APCs for antigen presentation and T cell priming (Figure 2).^12,38,39^ This approach offers several key advantages: (1) efficient engagement of a dense population of splenic APCs essential for initiating immunity; (2) robust systemic immune activation, leading to the generation of tumor-specific CD8^+^ T cells capable of targeting both primary and metastatic lesions; and (3) reduced off-target effects, as spleen-targeted biodistribution limits uptake by nonimmune tissues compared to liver-centric delivery platforms.^39^ Luo et al.’s study showed that spleen-targeting LNPs significantly upregulated antigen presentation pathways and T cell activation markers, correlating with robust cytotoxic T cell infiltration into PDAC tumors.^12^ This approach for improving mRNA vaccine efficacy is also exemplified by autogene cevumeran, which utilizes the immunological properties of the spleen for overcoming the immune-cold TME in PDAC.^13,18^ Furthermore, combining spleen-targeted delivery with systemic therapies, such as immune checkpoint inhibitors, may sustain effector T cell responses that further enhance therapeutic outcomes.

Irinotecan-loaded silicasomes synergize with spleen-targeting LNPs by inducing ICD at the primary tumor site, thereby triggering cancer cell death and release of tumor antigens that kickstart the cancer immunity cycle (Figure 2).^36^ These mesoporous silica nanoparticles, coated with a lipid bilayer, efficiently deliver irinotecan to tumor cells, triggering endoplasmic reticulum stress and apoptosis.^36^ The resultant release of damage-associated molecular patterns (DAMPs)—including calreticulin (CRT), high mobility group box 1 protein (HMGB1), and adenosine triphosphate (ATP)—activates local dendritic cells and promotes tumor antigen uptake.^7,36,37,40^ Co-delivery of irinotecan and the 3M-052 via the silicasome platform further enhanced antitumor immunity, as evidenced by increased CD8^+^ T cell infiltration and reduced regulatory T-cell populations in orthotopic KRAS-driven PDAC models.^40^ This combinatorial approach leverages ICD-induced antigen release alongside spleen-targeted immune priming to overcome PDAC’s intrinsically low tumor neoantigen burden, which amplifies the tumor-specific immune response (Figure 5).

The performance of transcriptomic analysis by Luo et al. corroborated the immune synergy discussed above, revealing robust and spatially distinct patterns of gene expression that underpin the complementary roles of silicasomes and lipid nanoparticles (LNPs) in reactivating the cancer immunity cycle.^12^ At the primary tumor site, bulk RNA sequencing demonstrated significant combined treatment upregulation of genes associated with antigen presentation (MHC class I and II), T cell activation and transcription initiation (e.g., T-bet and Eomes) (Figure 6). These findings reflect a favorable immune remodeling characterized by enhanced antigen processing, increased recruitment and activation of CD8^+^ T cells, and overall improved immunogenicity of the tumor microenvironment. This observed synergy reflects the generation of ICD that is induced by irinotecan (from the silicasome), innate immune activation by the TLR7 agonist, and expression of mutant KRAS provided by the transposon in cationic LNPs.

**Figure 6:**
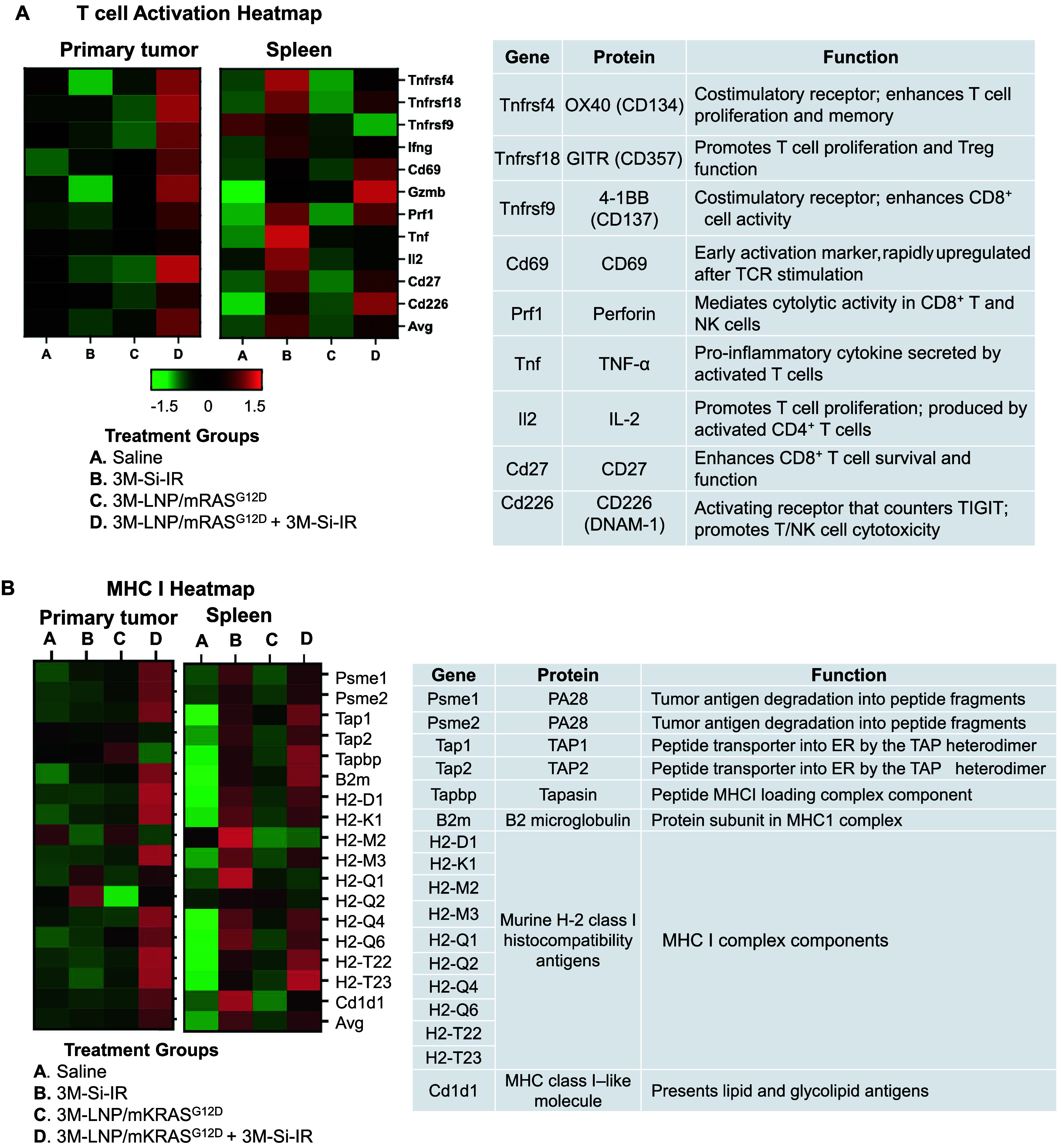
Transcriptomic profiling of dual-delivery immunotherapy in PDAC, corresponding to the experimental groups described in Figure 5.^12^ Treatment groups included: (A) saline, (B) irinotecan/3M-052 silicasome (3M-Si-IR), (C) KRAS∧G12*D*/3M-052 LNP (3M-LNP/mKRAS^G12D^), and (D) the combination of 3M-Si-IR with 3M-LNP/mKRAS^G12D^. Bulk RNA sequencing was performed on both primary tumor and spleen samples. (A) Heatmap of primary tumor genes associated with T cell activation demonstrates that the combination therapy (Group D) markedly upregulated T cell activation and effector function signatures in the primary tumor compared to saline and single-agent treatments. In contrast, the spleen also showed a rigorous response during treatment with 3M-Si-IR (group B). (B) Heatmap of primary tumor MHC class I pathway genes shows a similar pattern, with the combination therapy driving enhanced antigen processing and presentation at the tumor site. In contrast, transcriptomic analysis of the spleen revealed that 3M-Si-IR alone was sufficient to induce strong upregulation of MHC class I genes, with no further augmentation observed when combined with the KRAS^G12D^ LNP. Together, these data highlight that systemic immune priming is largely mediated by the silicasome platform, while maximal synergy of the dual-delivery approach occurring locally within the tumor microenvironment. Figures adapted with permission from ref 12 under a Creative Commons CC BY 4.0 license. Copyright 2025 The Authors.

Transcriptomic profiling of the spleen (performed postpublication) further revealed that irinotecan codelivery with 3M-052 by the silicasome is suffice for eliciting strong immune activation, particularly through upregulation of MHC I/II and T-cell activation genes (Figure 6). The addition of cationic mKRAS/TLR7 agonist LNPs did not further boost transcriptional events in the spleen, suggesting that the combination therapy achieves its maximal effect at the primary tumor site. Nonetheless, gross inspection of the spleen showed a dramatic reduction in its size in animals treated with either silicasomes alone or in combination with the LNP platform. This reduction in metastatic spread likely reflects localized tumor control combined with systemic immune surveillance. This illustrates the advantage of spatially targeted nanocarriers eliciting compartment-specific immune effects. Collectively, these findings emphasize the need to adapt nanomedicine immunotherapy not only by payload but also by anatomical site, underscoring the potential of integrating site-specific strategies for optimizing local as well as systemic antitumor immunity.

In summary, the dual-delivery strategy effectively bridges two major immunological bottlenecks in PDAC: insufficient antigen availability and poor immune priming. By coupling localized ICD-induced antigen release with targeted delivery of immunostimulatory signals to secondary lymphoid organs, the approach not only enhances intratumoral effector T-cell infiltration but also promotes the generation of systemic immune memory. Such synergy is particularly relevant for immunologically “cold” tumors like PDAC, where monotherapies with immune checkpoint inhibitors or chemotherapy alone have shown limited success. Moving forward, this integrated nanoplatform holds significant translational promise, offering a modular and adaptable framework for combining chemotherapy, adjuvants, and mRNA vaccines to elicit durable antitumor responses in PDAC, as further discussed below.

## 5 Discussion: Translating Preclinical Innovation into Broader Clinical Impact

The promise of immunotherapy in PDAC has remained largely unfulfilled due to the tumor’s profoundly immunosuppressive microenvironment, low mutational burden, and lack of effective immune activation in advanced diseases. However, a pivotal medical trial and to preclinical studies have begun to shift this paradigm by leveraging the precision of mRNA vaccines and the delivery potential of nanotechnology.^11–13^ These findings underscore the potential of integrating neoantigen-targeted mRNA with immune-activating adjuvants and chemotherapeutics using nanoparticle platforms to reprogram immune-resistant immune microenvironments plus restoring the cancer immunity cycle.

While the autogene cevumeran trial marked a breakthrough in demonstrating that approximately half of vaccinated patients mounted de novo neoantigen-specific CD8^+^ T cell responses—classified as “immune responders” whose recurrent-free survival was significantly extended—the efficacy was limited to early stage resected disease, free of metastases.^13,17^ The cost and complexity of generating individualized neoantigen vaccines, including whole-exome and RNA sequencing, remain barriers to scalability and broad clinical use.

In contrast, the preclinical studies alluded to provide critical mechanistic insights and translational guidance for extending the efficacy of immunotherapy to more advanced, metastatic stages of PDAC.^11,12^ The use of LNPs codelivering KRAS^G12D^ mRNA and the STING agonist cGAMP successfully reprogrammed the immunosuppressive liver niche in a metastatic PDAC model, promoting cytotoxic T-cell activation and tumor clearance. This codelivery approach triggered Type I interferon production, enhanced costimulatory molecule expression on liver-resident APCs, and generated cytotoxic CD8^+^ T cell responses capable of clearing liver metastases.^11^ Importantly, this immune response was durable, transferable, and effective prophylactically and therapeutically.

Further expanding on this strategy, the second preclinical study combined two distinct nanocarrier systems: irinotecan-loaded silicasomes to induce ICD at the primary tumor site, and spleen-targeting LNPs codelivering KRAS^G12D^ mRNA and the TLR7/8 agonist, 3M-052.^12^ This design links localized tumor antigen release with systemic T cell priming in the spleen, a central organ in adaptive immunity. The resulting synergy led to significant tumor regression and enhanced expression of antigen processing and T cell activation genes. These findings emphasize the importance of activating multiple arms of the cancer immunity cycle to overcome PDAC’s inherent immune resistance.

Given that these immune effects in the preclinical study are fundamentally driven by KRAS-derived neoantigens, it is critical to consider the mutational landscape of KRAS in PDAC and its implications for vaccine design. KRAS mutations represent one of the most defining and frequent genetic alterations in PDAC, occurring in >90% of cases. Among these, codon 12 mutations dominate, with KRAS^G12D^ being the most prevalent subtype, followed by KRAS^G12 V^ and KRAS^G12R^.^41,42^ These variants not only drive oncogenesis but also serve as promising neoantigen targets due to their cancer-specific expression and absence from normal tissues.

Preclinical and early clinical studies have further shown that KRAS^G12D^ possesses a relatively high degree of immunogenicity, eliciting CD8^+^ T cell responses in both murine models and human neoantigen screening platforms.^43,44^ This makes KRAS^G12D^ an ideal lead candidate for vaccine development. However, other recurrent mutations, such as KRAS^G12 V^ and KRAS^G12R^, have also demonstrated the capacity to serve as immunogenic epitopes, albeit with somewhat lower predicted or observed binding affinities to common MHC class I alleles.^45^ Still, these variants can activate T cells under optimized conditions, especially when paired with potent adjuvants like TLR agonists delivered via nanoparticles.^46,47^

Building on these mechanistic insights, recent clinical trials evaluating pooled KRAS peptide vaccines encompassing multiple codon 12 variants (G12D, G12 V, G12R, G12A, G12C, G13D) have provided further evidence of differential immunogenicity among these epitopes.^46^ For example, in a phase I study targeting high-risk individuals, G12 V elicited the strongest T-cell expansion (median 32.6-fold increase), while G12D was less immunogenic (median 7.6-fold).^48^ This underscores natural epitope variation and HLA binding significantly impacts immune potency.

In addition to relying on natural variation, synthetic strategies to enhance immunogenicity have emerged. Covalent modification of KRAS^G12C^–peptide/MHC complexes to create artificial neoantigens has been introduced: these hapten-modified epitopes can be targeted selectively, overcoming resistance in KRAS^G12C^ tumors.^49^ Complementing this, *in silico* sequence optimization and directed molecular evolution approaches have been applied to improve peptide–MHC binding and T-cell activation potential. Ng et al. (2018) demonstrated that rationally designed sequence modifications within KRAS G12 V and G13D epitopes can increase predicted MHC affinity and enhance immunogenicity in silico.^50^ More recently, Abdel Mouti (2023) explored chemical modification strategies to boost antigen recognition in KRAS^G12C^ tumors, representing a proof-of-concept for synthetic neoantigen engineering.^51^ Together, these advances suggest that combining structure-guided peptide design with machine learning–driven immunogenicity prediction could yield next-generation, optimized KRAS epitopes for mRNA vaccine development.

By targeting a panel of common KRAS mutant epitopes—particularly G12D, G12 V, and G12R—it may be possible to cover the vast majority of PDAC patients without requiring personalized sequencing and neoantigen prediction. This strategy holds promise for developing “off-the-shelf” mRNA vaccines, dramatically reducing cost, accelerating deployment, and extending immunotherapy access to patients with advanced or metastatic disease.

Complementing these mRNA vaccine strategies, the silicasome represents a clinically translatable nanocarrier platform designed to deliver chemotherapeutic agents that can synergize with immune-based therapies in PDAC. The lipid bilayer serves several critical functions: it stabilizes the silica core in physiological environments, reduces premature drug leakage, and shields the surface charge to minimize opsonization and nonspecific protein adsorption.^52,53^ When used for irinotecan delivery, this hybrid structure alters drug pharmacokinetics by prolonging circulation half-life and achieving controlled intratumoral release, thereby increasing therapeutic index.^53^ Preclinical studies have demonstrated that silicasome-encapsulated irinotecan significantly reduces gastrointestinal and hematologic toxicities compared to free or liposomal formulations by limiting systemic SN-38 exposure while maintaining high intratumoral drug concentration.^54^ These favorable safety and efficacy profiles support ongoing development of lipid-coated silica carriers as next-generation chemotherapeutic nanoplatforms with strong translational potential for PDAC and other solid tumors.

These combined insights suggest a practical framework for improving the impact of mRNA vaccination in PDAC. First, common KRAS mutations can be prioritized for off-the-shelf vaccine development. Second, targeted delivery to immune-privileged organs such as the liver and spleen can reverse tolerance and activate systemic immunity. Third, combining ICD-inducing chemotherapy with mRNA-adjuvant vaccination may synergize to restore the cancer immunity cycle.

Building upon this framework, recent progress in the clinical development of potent innate immune adjuvants—such as 3M-052 and cGAMP—further supports the translational feasibility of these nanomedicine-based immunotherapy strategies. 3M-052, a synthetic lipid-modified TLR7/8 agonist, has demonstrated potent local and systemic immune activation in multiple clinical trials, including as an immunomodulator in cancer therapy and as a adjuvant in protein-based vaccines for infectious diseases (e.g., NCT02556463, NCT04177355).^55,56^ Its hydrophobic tail, as demonstrated in our preclinical study, allows for stable incorporation into lipid carriers such as LNPs, enabling sustained immune stimulation while minimizing systemic cytokine toxicity.^57^ Similarly, 2′3′-cGAMP, a natural STING ligand, has shown favorable safety and pharmacodynamics in intratumoral and systemic delivery studies (e.g., NCT02675439, NCT03937141).^58^ However, its rapid degradation and limited membrane permeability have motivated nanoparticle encapsulation strategies that enhance delivery to APC and improve type I interferon signaling. Together, these adjuvants form clinically viable components of multifunctional nanovaccine platforms designed to reprogram PDAC’s immunosuppressive microenvironment.

Beyond PDAC, combining STING and TLR agonists has emerged as a powerful immunostimulatory strategy across multiple fields, including melanoma, triple-negative breast cancer, and infectious disease vaccines. Co-delivery of cGAMP with TLR7/8 or TLR9 agonists within nanoparticles has been shown to synergistically activate dendritic cells, enhancing type I interferon and pro-inflammatory cytokine production while driving robust CD8^+^ T-cell and humoral responses.^59–61^ In nanovaccine formulations, such as polymeric micelles, liposomes, and virus-like particles, this dual activation accelerates cross-presentation and durable memory formation.^59,60^ These insights directly inform PDAC immunotherapy, where immune exclusion and antigen presentation deficits are major obstacles. Integrating dual innate agonists within mRNA or chemotherapeutic nanocarriers thus represents a promising route toward multifunctional “immune rewiring” platforms capable of converting cold tumors into inflamed, T-cell–responsive lesions.

Although LNP- and silicasome-based immunotherapies show compelling preclinical efficacy, several translational challenges warrant consideration. Safety and metabolic tolerance remain primary concerns, as ionizable lipids and immune agonists (e.g., cGAMP, TLR7/8 ligands) can induce transient hepatic inflammation or cytokine release, necessitating careful dose and delivery optimization.^62–64^ Nanocarrier fate and clearance also pose risks—cationic LNPs may accumulate in the liver, while silica matrices require improved biodegradability to ensure safe elimination.^65,66^ Patient heterogeneity presents another limitation: variable HLA alleles, tumor clonality, and baseline immunity can lead to incomplete or absent responses.^18,67^ Additionally, off-target immune activation and innate sensing of exogenous RNA may cause systemic inflammation if not properly balanced.^68^ From a practical standpoint, manufacturing complexity and cost remain barriers. Producing dual-loaded GMP-grade LNPs or injectable silicasomes is technically demanding, and mRNA stability continues to constrain storage and distribution.^69,70^ Advances in lyophilization, thermostable lipids, and automated microfluidic encapsulation may help overcome these issues. Addressing these challenges through rational design, scalable manufacturing, and improved patient stratification will be essential to translating nanomedicine-enabled immunotherapy from experimental success to durable clinical benefit in PDAC.

While this review has primarily focused on systemically delivered nanovaccine strategies, local or in situ vaccination at the primary or metastatic site also warrants consideration.^71–73^ Direct intratumoral delivery offers the potential to convert the tumor itself into an active immunologic hub—enhancing antigen release, dendritic cell activation, and local T-cell priming precisely where immune suppression is strongest. This approach may be particularly valuable in PDAC, where the immune-excluded microenvironment, dense desmoplastic stroma, and poor lymphocyte infiltration limit the efficacy of systemic immunotherapy. However, implementing *in situ* PDAC vaccination presents practical challenges due to the pancreas’s deep retroperitoneal position and limited accessibility for direct injection. Encouragingly, advances in image-guided delivery, biodegradable polymeric or lipidoic nanocarriers, and stromal remodeling agents now make such strategies increasingly feasible.^71–73^ Integration of localized nanovaccine approaches with systemic mRNA–adjuvant platforms could ultimately enable dual-site immune activation, i.e., turning both the tumor and metastatic niches into sites of durable, self-sustaining antitumor immunity.

Together, these findings highlight the translational potential of nanomedicine platforms to fill the current therapeutic void in PDAC. By repurposing well-characterized oncogenic drivers like KRAS as immunogens, and codelivering immune agonists or chemotherapeutics with spatial precision, nanoparticles can overcome the immunologic and logistical barriers that have stymied prior efforts. Future work should aim to integrate these approaches into multiphase clinical trials and evaluate their combinatorial efficacy in both early stage and metastatic PDAC.

## 6 Conclusions

Taken together, the clinical and preclinical advances highlighted in this review support a transformative model for PDAC immunotherapy—one that leverages nanotechnology to reprogram immunosuppressive niches, enhance antigen presentation, and restore the cancer immunity cycle. The ability to deliver shared KRAS neoantigens in combination with potent immunostimulatory adjuvants provides a compelling path forward for treating both early and advanced PDAC. As nanocarrier platforms continue to evolve in specificity, scalability, and safety, these strategies stand poised to expand the reach of mRNA vaccines beyond personalized therapies, enabling broader, more equitable access. With rational integration of ICD inducers, adjuvant-loaded nanoparticles, and neoantigen payloads, it may soon be possible to convert even metastatic PDAC—a disease long considered immunologically untouchable - into one that is not only treatable but potentially curable through immune modulation.

## Vocabulary

Tumor microenvironment (TME): The cellular and molecular ecosystem surrounding tumors, including immune cells, stromal, cancer-associated fibroblasts, endothelial cells, extracellular matrix vascular, and immune components.
Antigen presentation: The process by which antigen-presenting cells (APCs) display peptide fragments bound to major histocompatibility complex (MHC) molecules on their surface for recognition by T cells.
Immunogenic cell death (ICD): A regulated form of tumor cell death that releases danger-associated molecular patterns (DAMPs) and tumor antigens to activate adaptive immunity.
Silicasome: A hybrid nanoparticle consisting of a mesoporous silica core coated with a lipid bilayer, designed for stable drug encapsulation and delivery.
Lipid nanoparticle: A nanoscale spherical particle composed of lipids that can encapsulate and transport therapeutic agents like mRNA and drug to targeted cells or tissues.
Immune checkpoint blockade: Antibody-based therapies that block inhibitory receptors such as PD-1 or CTLA-4, reinvigorating T cell function against tumors.
Bulk RNA sequencing: A transcriptomic analysis of RNA extracted from a mixed population of cells. This method measures average gene expression and provides global insights but lacks single-cell resolution.
